# Development and Performance Evaluation of Hybrid Iono-organogels for Efficient Impact Mitigation

**DOI:** 10.1021/acsaenm.4c00402

**Published:** 2024-09-27

**Authors:** Vincent Varanges, Vijay Kumar Rana, Valentin Phillippe, Pierre-Etienne Bourban, Dominique P. Pioletti

**Affiliations:** † Laboratory of Biomechanical Orthopedics, Ecole Polytechnique Fédérale de Lausanne (EPFL), Lausanne 1015, Switzerland; ‡ Laboratory for Processing of Advanced Composites, Ecole Polytechnique Fédérale de Lausanne (EPFL), Lausanne 1015, Switzerland

**Keywords:** organogels, ionogels, hybrid gels, impact mitigation, strain-rate sensitivity, viscoelasticity

## Abstract

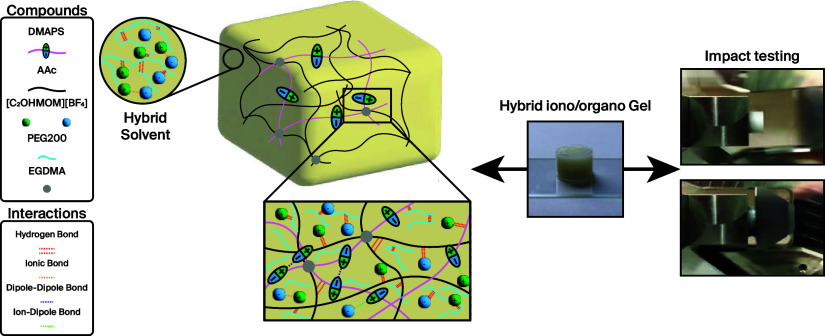

Dissipative materials are essential for mitigating impact in various automotive, aerospace, and sports equipment applications. This study investigates the efficiency of a novel hybrid iono-organogel in dissipating and absorbing impact energies. The gel consists of a covalently cross-linked poly(acrylic acid)-*co*-poly(zwitterionic (DMAPS)) in a hybrid solvent system composed of the ionic liquid [C_2_OHMIM][BF_4_] and the oligomer PEG200. The optimal solvent hybridization ratio for achieving the lowest deceleration during impact testing is 40 vol % of the ionic liquid and 60 vol % of PEG200. The gel exhibits efficient mechanical dissipative properties with a loss factor exceeding 0.5 when solicited under various dynamic conditions with this optimized ratio. Moreover, the gel demonstrates high strength and toughness, enabling it to withstand impacts without experiencing catastrophic failure. The developed gel presents stable mechanical properties over broad temperature (0–100 °C) and frequency (0.01–2000 Hz) ranges. It maintains its performance during successive impacts, thanks to its self-recovery abilities. The remarkable mechanical properties of the gel are attributed to the abundance of combined functional groups within the gel polymeric network. Indeed, reversible H-bonds, ion–dipole, and dipole–dipole interactions were observed in different studies to enhance mechanical performance. Their unique synergy effect in the developed hybrid gels held promise for better control of impact properties and durability in numerous dynamic applications.

## 1 Introduction

The risk of impact leading to destruction and injury is present in various engineering domains. Innovative technologies for impact mitigation, such as sports equipment,^1−3^ workplace safety,^4,5^ aircraft,^6^ and automotive industries,^7^ are actively sought. Current protective materials are typically tailored to minimize the risk associated with the most common impact scenarios at predefined energy levels. The specificity of the targeted material properties often makes them ineffective when facing alternative impact scenarios. In this context, polymeric materials are particularly interesting due to their viscoelastic behavior, resulting in adapted mechanical responses when subjected to various impact situations. By modulation of the viscoelastic behavior of these materials, it becomes feasible to fine-tune their energy dissipative properties, effectively minimizing stress transmission across a broad spectrum of impact scenarios.

Shear-thickening fluids have been and remain a subject of interest in developing energy dissipative materials.^8−12^ However, shear-thickening fluids exhibit two significant challenges to shape and process them due to their dilatant behavior and thickening effect, which decreases over time due to particle aggregation.^13,14^

Biological tissues such as adipose or skin can efficiently dissipate energy through viscoelastic behavior.^15−17^ Those tissues comprise a network of elastic fibers surrounded by a viscous fluid. Synthetic gels mimic the structure of biological tissues by infusing a solvent in a polymeric scaffold.^18−21^ Therefore, gels emerge as a potential candidate for impact-dissipative materials.^22−25^

Depending on the fluid’s nature, these gels can be categorized as hydrogels when water-based solvents are used, organogels when employing organic solvents or oligomers,^26−28^ and ionogels for systems involving ionic liquids (ILs).^29−31^ Each particular gel exhibits unique mechanical properties induced by various interactions between the polymeric scaffold and the fluid.

Organogels and ionogels demonstrate a multitude of dissipative interactions. These include the viscous dissipation resulting from the repetitive movement of the polymer fluid solvent within the scaffold and/or the associated rupture of hydrogen^22,32^ or dipole–ion bonds, particularly for ionogels.^33,34^ Each interaction within the material may be activated or ruptured at different energy thresholds.

This paper introduces a hybrid iono-organo gel designed for efficient impact mitigation across diverse energy intensities. By hybridization of the fluid solvent, we aim to capitalize on the energy dissipative mechanisms inherent to both organogels and ionogels. The gel is based on a poly(acrylic acid-*co*-3-dimethyl-(methacryloyloxyethyl) ammonium propanesulfonate) (p(AAc-*co*-DMAPS)) polymerized in the presence of a hybrid solvent composed of an imidazole ionic liquid [C_2_OHMIM][BF_4_] mixed with the oligomer PEG200. Our objective of this work is to optimize the composition of this hybrid solvent to maximize the impact mitigation and self-recovery capabilities of the resulting gel. The paper details the synthesis process and evaluates the gel’s performance through dynamic and static compression tests and through rheological and dynamical mechanical analyses. The results reveal that our hybrid gel not only withstands multiple impacts without losing its structural integrity but also shows exceptional impact mitigation, energy dissipation, and self-repairing properties. These characteristics indicate that fine-tuning the solvent hybridization ratio can significantly enhance the material’s impact resistance capabilities through reversible bond breakage and phase separation. This gel’s broad applicability and performance suggest its potential to improve safety features in diverse engineering fields.

## 2 Materials and Methods

### 2.1 Materials

The chemicals utilized in this study were acquired from the following sources: acrylic acid (AAc), [2-(methacryloyloxy)ethyl]dimethyl-(3-sulfopropyl)ammonium hydroxide (DMAPS), ethylene glycol dimethacrylate (EGDMA), alpha-ketoglutaric acid (α-KA), PEG200 from Sigma-Aldrich (St. Louis, MO, USA), and 1-(2-hydroxyethyl)-3-methylimidazolium tetrafluoroborate ([C_2_OHMIM][BF_4_], also referred to as IL in this study) from ABCR (Karlsruhe, Deutschland).

### 2.2 Hybrid Gel Synthesis

Hybrid gels were synthesized by using a one-step method. The precursor was prepared by dissolving the monomers AAc and DMAPS in the solvents (*C*_m_ = 3M), PEG200, and [C_2_OHMIM][BF_4_], with constant stirring at 60 °C for 2 h. Then, the cross-linker EGDMA (*C*_EGDMA_ = 0.25 mol % relative to monomer content) and the photoinitiator α-KA (*C*_α-KA_ = 1 mol % relative to monomer content) were added and dissolved in the above mixture. The solution was degassed at 60 °C for 10 min just before casting. Finally, the precursor was polymerized under an ultraviolet (UV) exposure chamber (UVACube 100 from Hönle) for a 5 min curing at 100 W. The ratio of each solvent was varied to assess their impacts on the mechanical properties of the hybrid gel.

### 2.3 SEM Images

The structure of the gel samples was analyzed via SEM (GeminiSEM from Zeiss (Oberkochen, Germany)) in high vacuum mode at an operating voltage of 3 kV. For this purpose, gels were soaked in deionized water for 48 h to replace the solvent and then freeze-dried for 48 h to remove the water. The dried samples were then frozen in liquid nitrogen and broken into pieces to be observed by SEM. Prior to observation, all samples were coated with a 10 nm layer of gold through plasma deposition.

### 2.4 Static Compression Test

Cyclic static compression tests were performed at a strain rate of 0.1 s^–1^ until 50% of strain was reached using an Instron E3000 linear mechanical testing machine (Norwood, MA, USA) equipped with a 250 N load cell. Compressive moduli were determined by linear regression until 5% of applied strain. The energy dissipation of the hydrogels was determined by the area enclosed in the hysteresis loop of the stress–strain curve.

### 2.5 Impact Test

Dynamic compression tests were performed using an Impetus from 4a Engineering (Traboch, Austria). The compression setup was used with a mass head of 1.75 kg. Impact testing was performed on cylindrical samples with a diameter of 15 mm and a thickness of 10 mm at an impact energy between 1 and 3 J, corresponding to an impact velocity of 1.07–1.85 m/s. Compressive moduli were determined by linear regression until 5% of applied strain. The energy dissipation of the hydrogels was determined as described above.

### 2.6 Rheological Shear Test

Shear tests were conducted utilizing an Anton Paar MCR 302e rheometer (Gratz, Austria). Measurements were implemented by using a parallel plate geometry with a diameter of 15 mm. The gels were glued on the disposable plate with cyanoacrylate to prevent sample slippage. The gel experienced a normal force of 1 N during the gap fine-tuning by the upper plate. Subsequently, the gel was put at rest for 1 min. Cyclic shear tests were performed at different strain rates from 0.01 to 1 s^–1^ by applying strain until 100%, with all measurements performed in triplicate.

### 2.7 Dynamical Mechanical Analysis (DMA)

DMA was performed on a Q800 analyzer (New Castle, DE, USA) in the linear shear mode. Rectangular samples with a 4 mm × 4 mm cross-section and a 2 mm thickness were characterized. First, a temperature ramp test consisting of a 3 °C/min heating ramp from 0 to 100 °C at 1 Hz and 1% strain was performed. Second, a time–temperature superposition (TTS) test was used, which is based on the measurement of the storage and loss modulus over a frequency range from 1 to 100 Hz at 1% strain and temperature ranging from 0 to 45 °C with a temperature increment of 5 °C. Then, TTS was performed according to the Williams–Landel–Ferry (WLF) equation at a reference temperature of 25 °C.

### 2.8 Oscillatory Rheological Measurements

Oscillatory rheological experiments were conducted on an Anton Paar rheometer MCR 302e (New Castle, DE, USA). The measurements were carried out in oscillatory shear mode by using a 15 mm diameter parallel plate geometry. Samples were prepared and mounted like those used for the rheological shear test. An amplitude sweep was performed at 10 rad/s between 0.01 and 50% of strain at 25 °C. Subsequently, a frequency sweep between 0.1 and 600 rad/s was recorded at 0.5% strain and 25 °C. The same sample preparation was performed for large amplitude oscillatory shear (LAOS) experiments. The strain amplitude varied from 0.1 to 100% at a frequency of 10 rad/s at a temperature of 25 °C.

## 3 Results and Discussion

### 3.1 Processing of the Hybrid Gels

**Figure 1:**
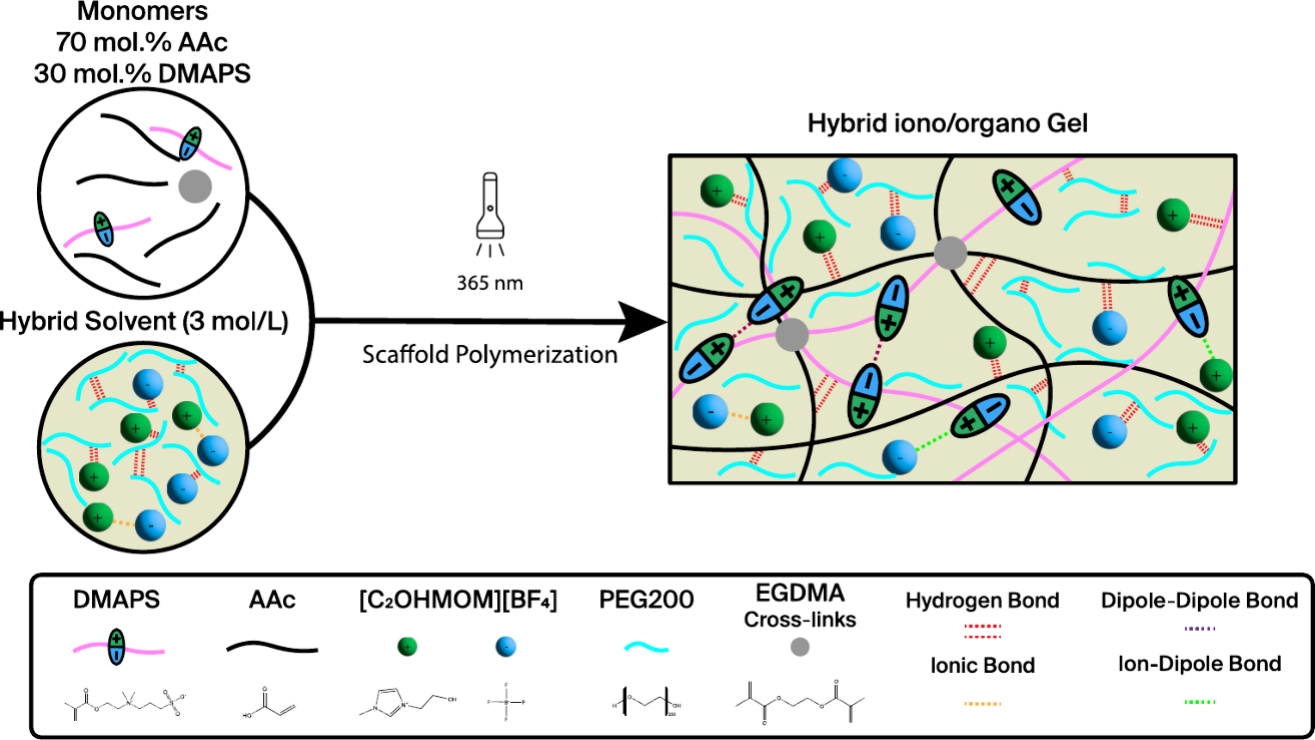
Schematic of the chemical structure of the hybrid iono-organo gel.

Our approach entailed a straightforward one-step photopolymerization of acrylic acid (AAc) and DMAPS in a 70:30 ratio, leveraging a 3 M concentration of monomer in a solvent blend composed of a [C_2_OHMIM][BF_4_] ionic liquid (IL) and PEG200. This particular mixture was selected to foster a range of dynamic and reversible chemical interactions and to explore their effect on the gel’s mechanical properties. The structural configurations and the intermolecular interactions within the hybrid gel are elaborated in Figure 1, which schematically represents the interactions among AAc, DMAPS, [C_2_OHMIM][BF_4_], and PEG200 through hydrogen bond, dipole–ion, and dipole–dipole interactions.

PEG200 is capable of forming a dense network of H-bond with the OH-groups of the AAc-*co*-DMAPS network and the group of PEG.^35^ Furthermore, hydrogen bonding occurs between the fluor atoms of [BF_4_] and the hydroxyl groups of the polymeric scaffold,^36^ allowing dispersion of the monomers before polymerization.^37^ The zwitterionic nature of DMAPS fosters as well unique molecular interactions, including dipole–ion interactions with the ionic liquid and dipole–dipole interactions among DMAPS molecules themselves.^33^

**Figure 2:**
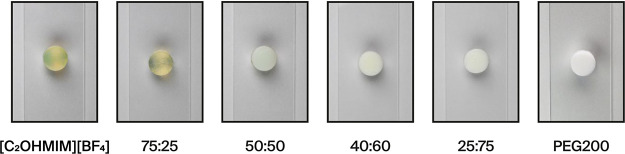
Pictures of the gel after polymerization as a function of the solvent hybridizations at a monomer concentration of 3 M.

To examine the impact of solvent hybridization, various gels were synthesized by varying the proportions of [C_2_OHMIM][BF_4_] ionic liquid (IL) to PEG200 in mixtures of AAc and DMAPS, resulting in different gel types: ionogels, organogels, and hybrid gels, as illustrated in Figure 2.

When there is an excess of IL, the synergistic interactions within the hybrid gel result in a quasitransparent gel with a homogeneous structure. With an excess of PEG200 relative to the IL, the gel turns from transparent to opaque with a whitish color. This is due to the poor compatibility between PEG200 and DMAPS, which causes phase separation. This separation enhances dipole–dipole interactions among DMAPS’s zwitterionic groups.^38^ Despite the phase separation, the gel retained its shape without a leakage.

**Figure 3:**
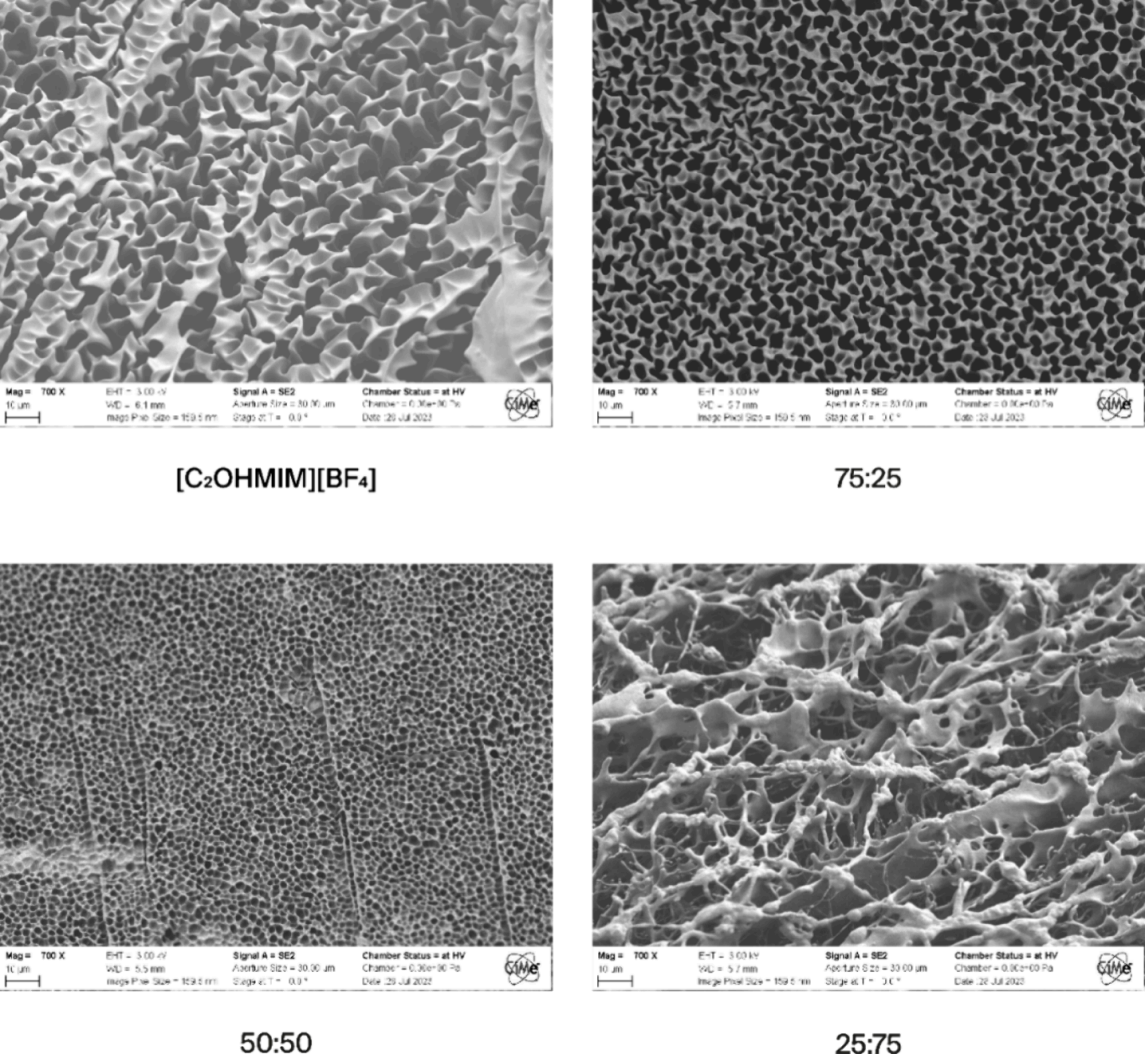
SEM images with a magnification of 700 and after solvent removal of the iono-organogel scaffolds obtained with different initial solvent hybridization ratios.

The gel’s polymeric scaffolds can be observed in the SEM images of Figure 3.

The pure ionogel and the 75:25 hybrid gel exhibit homogeneous scaffolds. However, increasing the PEG200 content leads to the formation of additional walls and spherical microstructures in the polymer scaffold, as seen in samples with equal volumes of IL and PEG200 (50:50). Furthermore, when the hybrid ratio is adjusted to 25:75, the scaffold becomes discontinuous, characterized by large aggregates with nonhomogenous walls. This likely results from the intensified interactions among DMAPS polymer chains driven by the formation of dipole–dipole bonds. Additionally, it was not possible for the SEM observation to substitute the solvent in the full organogel (100 vol % PEG200), as immersion in water for 2 days led to the complete dissolution of the gel. This outcome is attributed to the presence of domains within the gel due to phase separation. These domains are not effectively cross-linked with each other. This lack of interdomain cross-linking leads to the gel’s dissolution. This behavior was not observed in other compositions, indicating that the ionogel is critical in facilitating cross-linking within the hybrid gel. The ionic liquid helps maintain the structural integrity of the gel by promoting cross-linking between different domains through increased solubility of each monomer among the ionic liquid.

In conclusion, the hybridization of the gel’s solvent led to an increase in the type and the number of chemical interactions between the solvents and the monomer and different polymer microstructures.

### 3.2 Mechanical Properties of the Hybrid Gels under Compression

**Figure 4:**
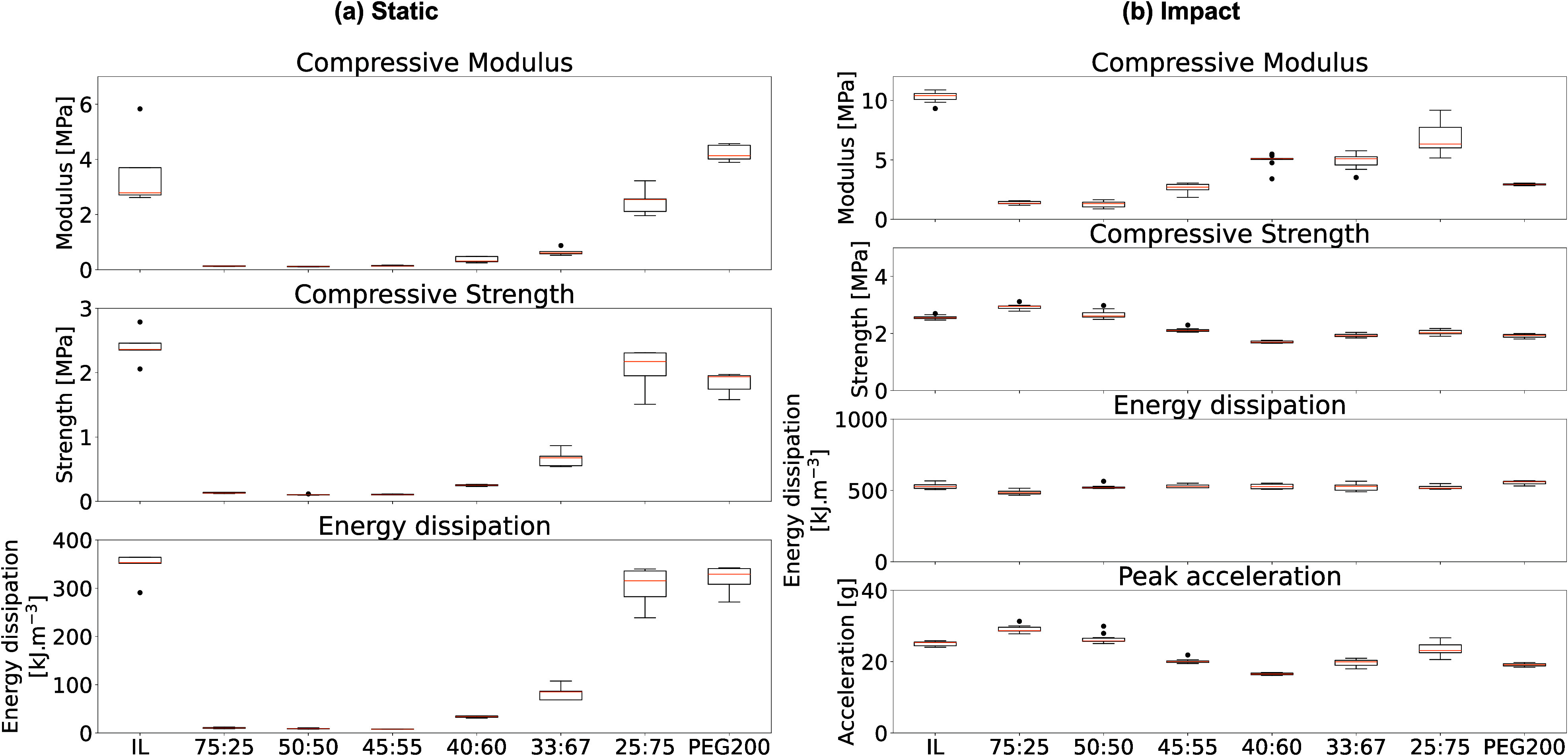
Effects of solvent hybridization on mechanical properties (a) under static compression conditions and (b) under impact conditions.

We explored solvent hybridization’s influence on our gels’ compressive properties. The outcomes of this analysis are presented in Figure 4a for the static tests.

**Figure 5:**
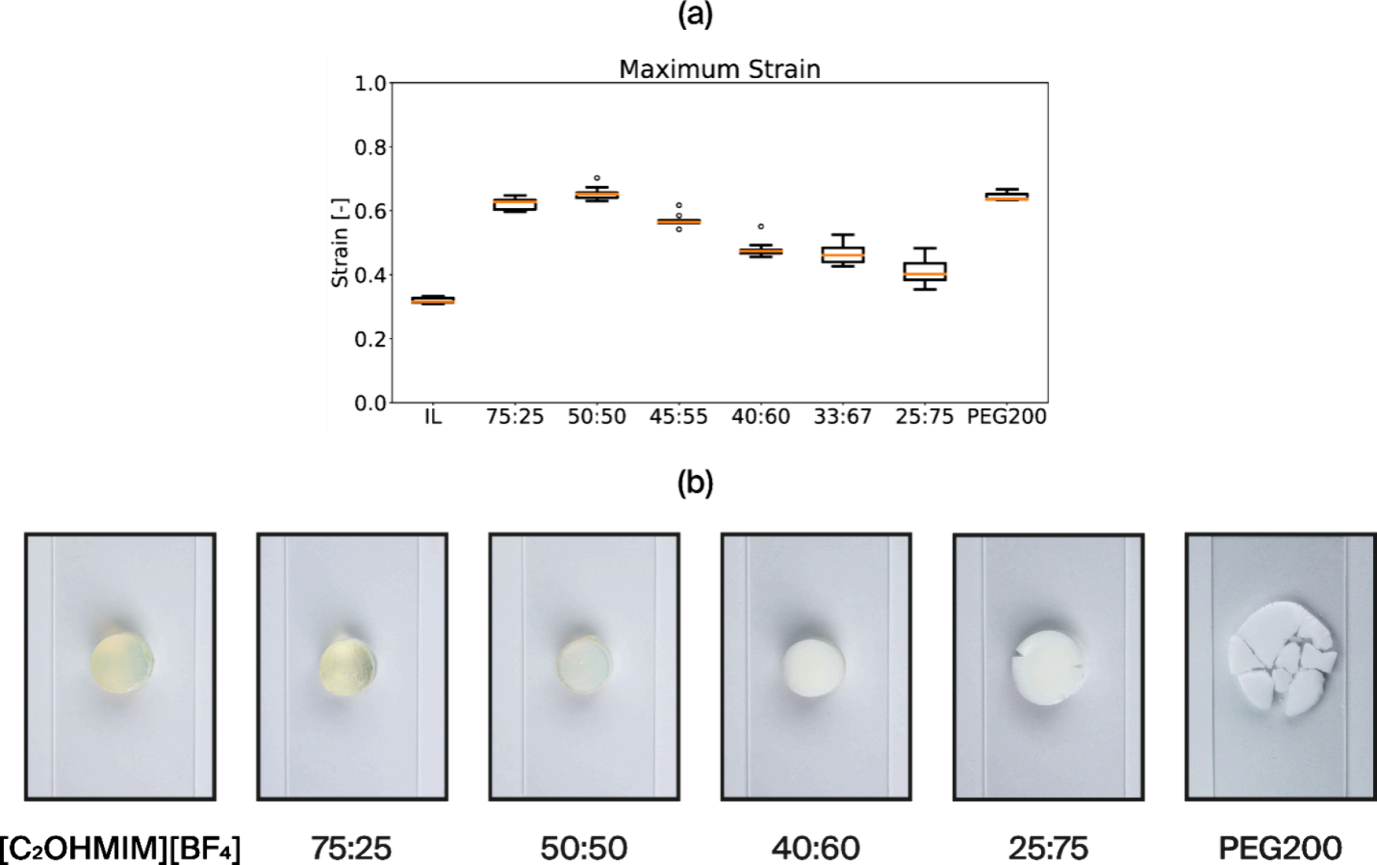
(a) Maximum strain at 1 J impact. (b) Pictures of the hybrid gels after a single impact at 1 J impact.

We noticed a significant decrease in mechanical properties when hybridizing the solvent of the gel. This behavior stemmed from the low compatibility between the DMAPS and the PEG200, which decreased the compressive modulus from 3.53 MPa for the ionogel to 0.13 MPa at 25 vol % of PEG200. A substantial increase in energy dissipation was noted at high concentrations of PEG200, ranging from 34 kJ m^–3^ at 60 vol % to 302 kJ m^–3^ at 75 vol %. Gels containing 75 vol % or more of PEG200 exhibited notable energy dissipation due to their brittle nature. Notably, the creation and propagation of cracks caused by the phase separation within these gels play a major role in energy dissipation. However, this mechanism results in irreversible damage to the gel with only a minor portion of the dissipated energy stemming from reversible bond breakage. Overall, gels with less than 75% PEG200 by volume did not break and could withstand up to 50% strain without failure. Figure 5a shows the maximum strain the gels experienced, while Figure 5b shows their morphology after the impact.

The gels were then assessed for their impact mitigation capabilities. Figures 4b and 5 illustrate the effects of solvent hybridization between the IL ([C_2_OHMIM][BF_4_]) and the PEG200 on the mechanical properties of the polymerized hybrid gels. Under dynamic loading, we observed a pattern similar to that seen with static loading. Initially, adding PEG200 to the IL reduced the compressive modulus, suggesting that PEG200 interferes with the stiffness typically provided by the dipole–ion interactions between the IL and the polymeric scaffold. For instance, the compressive modulus dropped from 10.3 MPa in pure ionogels to 1.5 MPa when 25 vol % of PEG200 was incorporated into the hybrid gel. This indicates that the IL interacts more with PEG200 than does the polymeric network, resulting in decreased stiffness. However, as the proportion of PEG200 increased, phase separation occurred, leading to a denser phase of polymerized DMAPS. This change increased the gel’s stiffness from 1.5 MPa at 25 vol % PEG200 to 6.8 MPa at 75 vol % PEG200. In contrast, gels with more than 75 vol % PEG200 exhibited poor structural integrity, rendering them brittle. The separated phases of polymerized DMAPS aggregates could lead to the complete fracture of the material after a single impact, as seen in Figure 5b. These phases provide sites where cracks may initiate and propagate, therefore contributing to energy dissipation.

The measured reductions in the compressive strength and the peak acceleration indicated an enhanced ability to efficiently dissipate the energy from an impact. The hybrid gel composed of 40 vol % [C_2_OHMIM][BF_4_] and 60 vol % PEG200 demonstrated the most promising performance for impact dissipation. Despite experiencing a peak acceleration of 16.6 g, this gel remained structurally intact without any postimpact damage. This behavior certainly stems from the interactions between the compounds and also benefits from phase separation, which enhances the efficient dissipation of energy. Moreover, due to the incompressibility of the liquid enclosed by the polymeric scaffold, most gels have a Poisson ratio approaching 0.5. Therefore, they adapt their impacted surface according to the incoming load. The optimized hybrid gel (40:60) offers an optimum compromise of the reactive polymer–solvent interactions in a homogeneous phase-separated microstructure.

**Figure 6:**
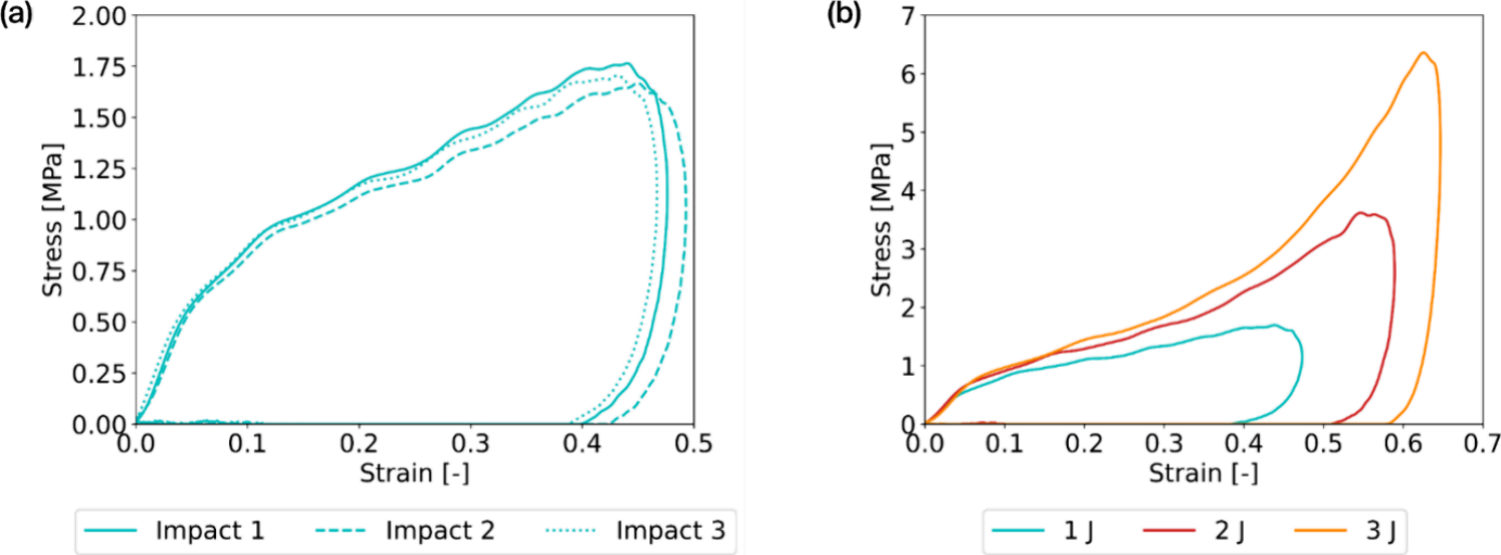
Dynamic characterization of the optimized hybrid gel composed of 40 vol % in [C_2_OHMIM][BF_4_] and 60 vol % in PEG200: (a) effect of impact repetition on stress–strain curves and (b) effect of energy of impact on stress–strain curves.

The (40:60) optimized hybrid gel was also impacted three times with 1 min of rest between each impact. The resulting stress–strain curves are displayed in Figure 6a.

The gel recovers its initial mechanical properties after each impact. This restorative ability can be attributed to the reversibility of a significant proportion of the chemical bonds formed within the gel, namely, hydrogen, ionic, dipole–dipole, and ion–dipole bonds, as displayed in Figure 1. They undergo breakage under loading and subsequently reform after resting, contributing to the gel’s capacity for self-recovery. Moreover, this gel can withstand successive large deformations without polymeric network rupture.

In addition, this optimized gel (40:60) was subjected to three levels of impact energy, leading to different deformation rates: 57, 90, and 118 s^–1^. Figure 6b displays the obtained stress–strain behaviors, revealing the gel’s capacity to dissipate energy over various impact energies. The optimized gel could adapt to the incoming impacts through its viscoelastic properties.

### 3.3 Mechanical Properties of Hybrid Gels under Shear

**Figure 7:**
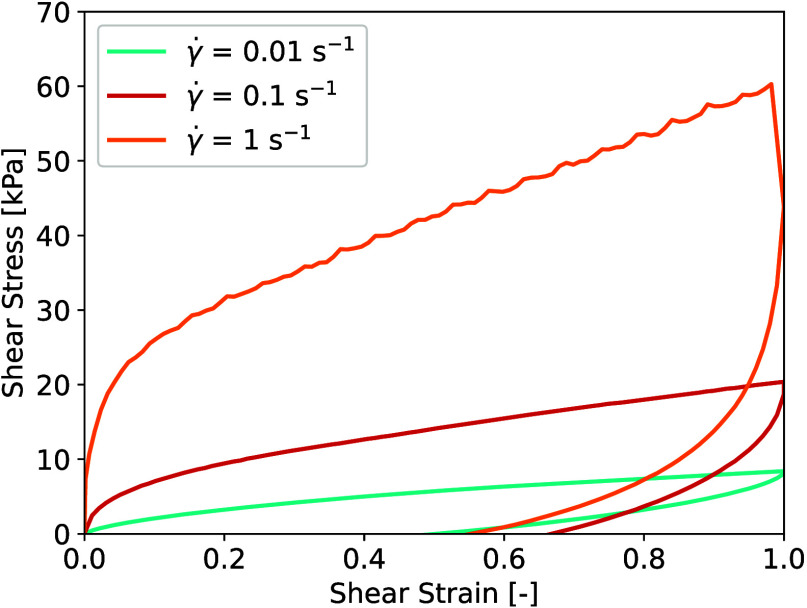
Shear testing of the hybrid iono-organogels composed of 40% [C_2_OHMIM][BF_4_] and 60% PEG200 at three different shear rates: 0.01, 0.1, and 1 s^–1^.

As materials experience compression and shear under an impact, the dissipative properties under shear loading are essential for enhancing impact mitigation.^39^ In this context, the shear properties of the 40:60 hybrid iono-organogel, composed of 40 vol % IL and 60 vol % PEG200, were evaluated. Using a rheometer, measurements were conducted at three different shear rates. The cyclic shear stress–shear strain curves, as depicted in Figure 7, demonstrate a pronounced sensitivity to the shear rate, with mean peak stress increasing from 7.9 to 46.1 kPa when passing from a shear rate of 0.01–1 s^–1^. Furthermore, the gel’s significant dissipation capabilities are illustrated by the large observed hysteresis and energy dissipations increasing from 3.6 to 29.2 kJ m^–3^ at the highest rate (Table 1).

**Table 1:** Shear Properties of the Optimized Hybrid Iono-organogel Composed of 40% [C_2_OHMIM][BF_4_] and 60% PEG200

shear rate/shear properties	0.01 s^–1^	0.1 s^–1^	1 s^–1^
shear modulus [kPa]	16 ± 2	39 ± 10	96 ± 27
peak stress [kPa]	7.9 ± 0.7	18.0 ± 2.3	46.1 ± 10.9
energy dissipation [kJ m^–3^]	3.6 ± 0.6	10.3 ± 2.7	29.2 ± 6.4

We observed a substantial disparity between the compressive and shear moduli at a deformation rate of 0.1 s^–1^, with values of 360 and 39 kPa, respectively. This discrepancy indicates a pronounced tendency of the material to undergo shear deformation. The shear characterization highlights the gel’s ability to dissipate energy under various loading modes. This capability stems from the formation of a dynamic molecular network, which is rich in diverse and numerous intermolecular forces while allowing free movement of the two solvents. These findings indicate strong viscoelastic behavior in the hybrid gel. Using DMA, we can further investigate the gel’s dissipative properties under shear sandwich conditions, measuring the loss factor (tan δ) during temperature and frequency sweeps.

A high loss factor means efficient energy dissipation and stress reduction. In this study, we used a dynamic mechanical analyzer to investigate the dissipative properties of the hybrid gel under shear sandwich loading. The gel was subjected to a temperature ramp from 0 to 100 °C to evaluate its energy dissipation efficiency across temperatures while maintaining mechanical properties.

**Figure 8:**
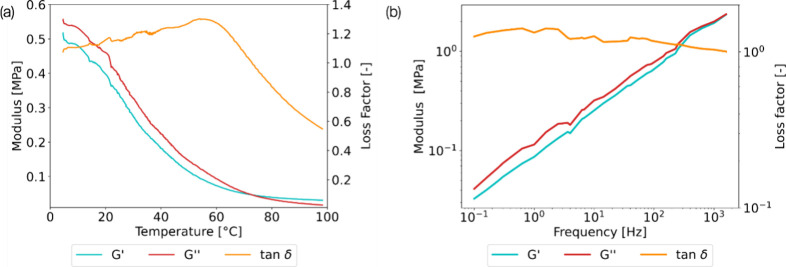
DMA testing of the optimized hybrid iono/organogels composed of 40% IL and 60% PEG200 (a) temperature ramp between 0 and 100 °C with storage modulus (*G*′), loss modulus (*G*″), and loss factor (tan δ) displayed. (b) Frequency dependence of storage modulus (*G*′), loss modulus (*G*″), and loss factor (tan δ) after time temperature superposition at the reference temperature of 25 °C.

The storage modulus displayed in Figure 8 was consistently above 0.1 MPa until 53 °C. The gel maintained a loss factor exceeding 0.5 over the temperature range. Notably, the loss factor was around 1 between 0 and 60 °C, indicating that the gel exhibited behavior akin to both a solid and a fluid while maintaining high storage and loss modulus values—ensuring its ability to retain its shape over time. The gel dissipative capabilities were slightly reduced above 60 °C. Viscous residuals were discovered when removing the sample from the DMA. At this temperature, the solvent leaks, causing a loss of its viscoelastic properties.

We further used time-dependent temperature superposition (TTS) with DMA to evaluate the viscoelastic properties of the optimized gel over a wide range of frequencies. According to the trend of the storage modulus in Figure 8b, similar to typical viscoelastic materials, the gel exhibited a stiffer response as the frequency of the mechanical loading increased. Considering the loss factor, our gel displayed strong energy dissipation capabilities over a range of frequencies, from 0.01 to 1000 Hz. This adaptability is attributed to the diverse energy dissipation mechanisms inherent to the hybrid gel.

**Figure 9:**
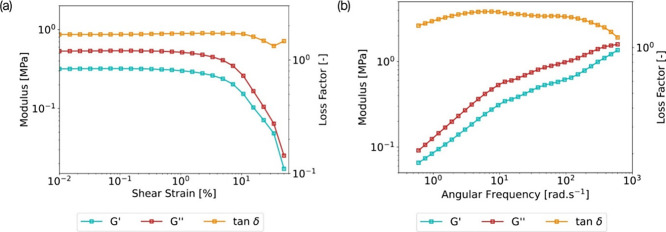
Rheological testing of the optimized hybrid iono-organogels composed of 40% IL and 60% PEG200: (a) amplitude sweep at 10 rad/s and 25 °C for oscillating shear strain ranging from 0.01 to 50%; (b) frequency sweep from 0.6 to 600 rad s^–1^ at 0.5% of shear strain and 25 °C.

Rheological tests were conducted to characterize the gel’s shear properties further. An amplitude sweep was performed to evaluate the gel’s linear viscoelastic region (LVER) under shearing conditions. According to the results in Figure 9a, the limit of the LVER was 1%, and the gel consistently exhibited a loss factor superior to 1 across the entire range of strains, therefore displaying high energy dissipation. To ensure testing in the LVER while performing a frequency sweep, a strain of 0.5% was selected for further testing.

Figure 9b showcases the frequency sweep results of the gel, confirming a quasi-constant loss factor across a wide range of frequencies. However, regarding the storage modulus *G*′, an increasing trend was observed with the rise in angular frequency. This behavior highlights the gel’s viscoelastic nature, demonstrating its capability to adapt to varying shear strain rates by exhibiting an ascending *G*′ in response to increasing frequencies.

**Figure 10:**
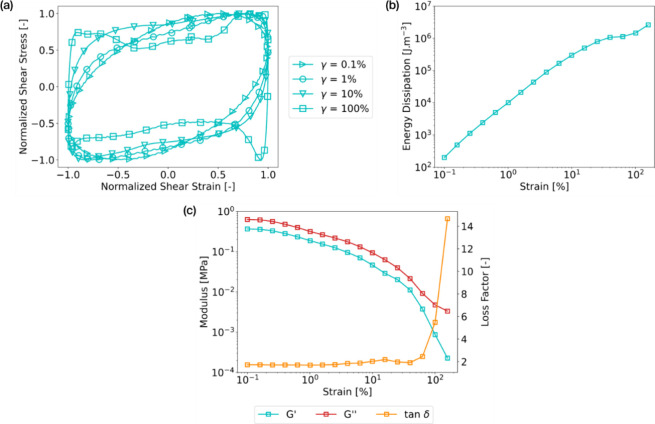
Large amplitude oscillation shear experiment on hybrid gels composed of 40% of [C_2_OHMIM][BF_4_] and 60 vol % of PEG200 at 10 rad/s and 25 °C: (a) scaled Lissajous plots for varying strain amplitude from 0.1 to 100%; (b) energy dissipation as a function of strain amplitude during the LAOS experiment ranging from 0.1 to 100% corresponding to the hysteresis areas; (c) storage modulus (*G*′), loss modulus (*G*″), and loss factor (tan δ) as a function of strain amplitude during LAOS.

Under impact loading, the gel is subjected to a large and rapid deformation beyond its linear viscoelastic region. Therefore, it is the nonlinear properties that govern the rheological response of the gel. We employed the large amplitude oscillatory shear (LAOS) method to evaluate them. Lissajous plots represent the normalized stress vs the normalized strain cycles obtained from each strain sweep test, with strains varying from 0.1 to 200%. Each plot corresponds to a specific viscoelastic response at a given strain. Analysis was constrained to within a 200% strain limit. This limitation was imposed based on prior testing experiences, which revealed a decoupling issue between the rotating measurement system and the gel despite gluing the interface. According to the Lissajous plots in Figure 10a, the gel tended to stiffen with increasing strain cycles. In the LVER, the Lissajous plots were elliptical as a function of shear strain, as can be observed for cycles at 0.1 and 1% of strain. Increasing the strain, the Lissajous plot tends to distort into an oblong shape, particularly at 10% strain. A peak in shear stress at maximum and minimum shear strains is noticed. This behavior is characteristic of a shear-stiffening material.^40,41^

The Lissajous plots are the hysteresis of the shear stress as a function of shear strain. Therefore, we obtain the dissipated energy during each cycle by integrating the non-normalized shear stress over the non-normalized shear strain (Figure 10b). There is a constant increase in energy dissipation according to the strain, meaning that under the shear test, the gel changes its viscoelastic response. This behavior is likely attributed to the breakage of the reversible bonds present in the gel. Figure 10c illustrates the gel viscoelastic response during the LAOS experiment strain sweep. Our primary focus of our work lay in the region of nonlinear viscoelasticity, where we observed a decline in both the storage and loss moduli. This behavior was characteristic of the nonlinear viscoelastic region. At around 60% of strain, a sol–gel transition led to a fluid-like behavior of the hybrid gel, leading to a strong increase in the loss factor reaching 15 at 200% of strain.

### 3.4 Generalized Understanding of the Mechanisms Inducing Energy Dissipation in Iono-organogel

The comprehensive characterization of the dissipative properties of the iono-organogel has yielded a nuanced understanding of the various dissipative mechanisms and their influence on the gel’s mechanical properties.

Each material component contributes to the formation of interactions capable of dissipating energy, which is a key advantage of using gel-like materials composed of two different solvents. These interactions form a dense and varied network. Among these mechanisms, viscous flow is the most significant process for dissipating energy within the iono-organogel. It also promotes a high Poisson ratio, thereby enhancing the deformability and adaptability. Viscous flows are driven by the sliding of polymer chains past each other and the movement of the ionic liquid within the polymeric matrix, providing resistance to flow. This phenomena involves solvent movement along networks, such as pDMAPS and pAAc.^42^ Costa et al. highlighted the importance of this type of dissipation, noting that it can lead to local temperature increases and viscosity changes in response to shear strain, direct consequences of the interplay between energy and momentum equations.^43^

Additionally, chain reptation, which relies on the movement of polymer chains, plays a crucial role in energy dissipation. This process occurs through internal friction between the polymer fluids and the polymer network, leading to entanglement. Coming from the presence of the polymer fluid PEG200, the iono-organogels exhibit a whole chain reptation polymer network leading to strong energy dissipation.^32^ The segment oscillation of cross-linked polymers also contributes to energy dissipation in the iono-organogel. It refers to the mobility of polymer chains, specifically pAAc and pDMAPS, during loading. The oscillations of these polymeric networks offer low to medium energy dissipations, as they are in a high-elastic state and thus return a large portion of the energy.

Moreover, the poor solvation of DMAPS inside PEG200 and the strong interactions between DMAPS polymer chains from dipole–dipole interactions induce phase separation. The formation of such domains enhances the gel’s ability to distribute stress and improve energy dissipation while also increasing stiffness, as demonstrated by Wang et al.^34^ However, this phase separation can also make the gel more susceptible to fracturing due to the creation of distinct domains at high concentrations, as displayed in Figure 5.

Another significant family of dissipative mechanisms involves bond breakage. Within this category, two types can be distinguished: irreversible and reversible interaction breaking. In the iono-organogel, irreversible bond breakage primarily results from the disruption of covalent bonds.^44−46^ These bonds are formed between polymeric monomers through UV polymerization and chemical cross-linking. These robust bonds confer high stiffness and strength to the gel. When these polymer chains fracture, the mechanical energy stored within them is dissipated. However, such fractures lead to irreversible damage to the polymer networks. The components of the iono-organogels are capable of forming multiple reversible bonds, including ionic bonds, hydrogen bonds, dipole–dipole interactions, and ion–dipole bonds (see Figure 1).^33,47−49^ Although these bonds play a crucial role in the energy dissipation of the gel, they do so at a lower magnitude compared to those in viscous flow and chain reptation. However, these reversible interactions are vital for enhancing the recovery capabilities of the gel, enabling it to regain its original structure and functionality after deformation, as demonstrated in Figure 6.

Future work should investigate these dissipation mechanisms individually to understand their complex interrelationships better. Additionally, research should aim to discern how each mechanism contributes to the gel’s overall energy dissipation and quantify these contributions. Such research should help fine-tune the gel’s composition to achieve the optimal interactions for mitigating the specific impact scenarios to which they are subjected.

## 4 Conclusions

Our research on gels introduces a novel approach for impact mitigation by tailoring both the polymer network and the imprisoned solvent, highlighting the resulting responses to various specific loading conditions.

This study presents an innovative hybrid iono-organogel synthesized through a one-step photocopolymerization process. After testing various compositions, we finalized a hybrid gel containing 40 vol% ionic liquid and 60 vol% PEG200, with constant AAc and DMAPS monomer concentrations. This optimal gel exhibits a strong energy dissipation and remarkable self-recovery capabilities. These properties make it an excellent choice for applications that require resilience against multiple impacts.

The gel can effectively dissipate energy across diverse deformation rates, temperatures, and loading modes due to synergies between reversible chemical interactions, in situ generated microstructures, and internal flows of polymer networks and contained liquids. Furthermore, phase separation within the gel emerges as a crucial factor in its efficient energy dissipation mechanism. The segregation of components leads to distinct phases that interact under stress, enabling the gel to absorb and dissipate energy through enhanced dipole–dipole interactions and increased interfacial dynamics. This phase separation contributes to the gel’s mechanical robustness and optimizes its energy absorption characteristics under various impact conditions. This inherent flexibility in dissipating energy across varying frequencies and time scales underscores its potential for numerous practical applications. Notably, the material’s sensitivity to strain rates, ranging from 0.1 to 150 s^–1^ under both compression and shear loadings and its response to frequencies between 0.1 and 1000 Hz, suggests its promising use in protective equipment.
